# Perceptions of ethnoveterinary medicine among animal healthcare practitioners in South Africa

**DOI:** 10.4102/ojvr.v91i1.2138

**Published:** 2024-07-31

**Authors:** Rendani V. Ndou, Simeon A. Materechera, Mulunda Mwanza, Wilfred Otang-Mbeng

**Affiliations:** 1 Centre of Animal Health Studies, Faculty of Natural and Agricultural Sciences, North-West University, Mmabatho, South Africa; 2 Department of Indigenous Knowledge Systems Centre, Faculty of Natural and Agricultural Sciences, North-West University, Mmabatho, South Africa; 3 School of Biology and Environmental Sciences, Faculty of Agriculture and Natural Sciences, University of Mpumalanga, Mbombela, South Africa

**Keywords:** African traditional medicine, ethnoveterinary medicine, indigenous knowledge systems, animal health care, farmers, state veterinary, integrated health care

## Abstract

The South African government recognises the value of traditional healing and supports an integrated healthcare system. In the veterinary field, successful integration depends on the perceptions of different animal healthcare practitioners (AHCPs). This study aimed to determine the perceptions of ethnoveterinary medicine among state-employed veterinarians and animal health technicians in the North West province. A mixed model questionnaire and non-probability sampling gathered quantitative and qualitative data from 53 participants. Statistical and thematic analysis processes were applied to quantitative and qualitative data, respectively. Overall, 77.4% of participants were aware of ethnoveterinary medicine practices. Approval of the use of ethnoveterinary medicine by farmers was above-average at a mean of 3.57, standard deviation (s.d.) = 1.016 on a 5-point Likert scale. However, 65.9% doubted the effectiveness of ethnoveterinary medicine, which suggests tolerance but no appreciation of its value. The Chi-square test revealed a significant relationship between district municipality of employment and awareness at χ^2^ = 17.490, *df* = 3, *p* = 0.001. Thematic analysis of comments showed that some participants perceived ethnoveterinary medicine as unscientific and interfering with Western veterinary medicine, while some considered it a good complement to Western veterinary medicine. The study also found that 20.8% of AHCPs use ethnoveterinary medicine to treat certain diseases. In conclusion, the lack of appreciation for ethnoveterinary medicine among AHCPs may hinder government-proposed integration, emphasising the need for programmes to improve perceptions among these stakeholders.

**Contribution:**

This is first such study to report on perceptions of ethnoveterinary medicine among AHCPs, and assess their readiness for an integrated animal health system.

## Introduction

African ethnoveterinary medicine (AEVM) is derived from African traditional medicine (ATM), which is part of African Indigenous Knowledge Systems (Semali & Kincheloe 2002; Wanzala et al. 2005). Like any other form of traditional medicine, these practices have survived the test of time and even survived the era of colonisation in many African countries. In South Africa, this system was able to survive even though it was labelled as superstition and magic, and was legally banned. However, after the democratic elections of 1994, the South African government recognised the role played by traditional healing in the lives of South Africans and sought to right the wrongs of the past by legalising and regulating traditional practices (Mokgobi 2012). Traditional medicine is particularly important in rural areas, where access to appropriate and affordable primary healthcare is difficult or impossible (Pefile 2005).

The South African government recognises the value of traditional medicine, including ethnoveterinary medicine (EVM), especially as a primary healthcare solution, and supports integrating Western and traditional healthcare systems (Pefile 2005). The recognition of the value of traditional medicine led to the development of a legislative framework that includes the *Traditional Health Practitioners Act 22 of 2007* (the THPA), and the draft policy on ATM for South Africa, 2008. However, the success of any integration endeavour depends on the perceptions of stakeholders in the different health fields. In the South African animal health sector, veterinarians and para-veterinarians would be critical in the process of integrating Western veterinary medicine with EVM and preservation of knowledge of EVM. While many studies have been undertaken to evaluate the perceptions of Western-trained human-health practitioners toward ATM (Hopa, Simbayi & Du Toit 1998; Mokgobi 2012; Nemutandani, Hendricks & Mulaudzi 2016; Peltzer et al. 2008), studies on the perceptions of Western-trained animal healthcare practitioners (AHCPs) have been neglected.

This study aimed to establish the perceptions of ethnoveterinary practices among Western-trained state-employed AHCPs. The study thus determined the readiness of AHCPs to integrate Western medicine and EVM.

## Materials and methods

### Study area

**FIGURE 1:**
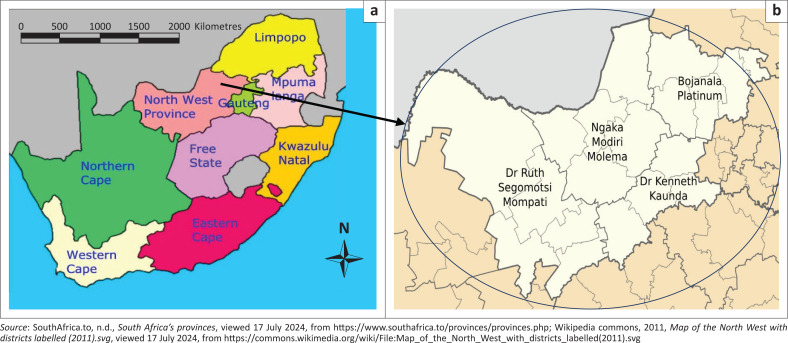
Map of South Africa on the left showing the position of North West province and on the right is the map of North West province and its four District Municipalities.

The study was conducted in the North West province of South Africa, which is bordered by Botswana to the north, the Kalahari Desert to the west, Gauteng province to the east, and the Free State to the south. The province covers 104881.67 km^2^ and has a population of 3 509 953 people. It has more sunny days and higher average rainfall per year than the South African average, making it advantageous for agriculture (North West Province 2024). The North West province is divided into four district municipalities, which are further subdivided into 18 local municipalities. The four district municipalities of the North West province are, Bojanala Platinum District Municipality; Dr Kenneth Kaunda District; Dr Ruth Segomotsi Mompati District; and Ngaka Modiri Molema District Municipality (Figure 1). The province is known as the Platinum province for its abundance of the underground metal (North West Province 2024).

Ngaka Modiri Molema and Dr Ruth Segomotsi Mompati are the two poorest rural district municipalities in the North West province. Dr Ruth Segomotsi Mompati District Municipality is considered the rural hinterland of the province, with vast rural areas containing scattered, small, low-level urban nodes (North West Province 2015). The majority of economic activities in the North West province are concentrated in the southern region between Potchefstroom and Klerksdorp in the Dr Kenneth Kaunda District Municipality, as well as Rustenburg in the Bojanala Platinum District (North West Province 2013). Mining is the largest contributor to the economy of the North West province, representing almost a quarter of South Africa’s mining industry as a whole. The province produces gold from Orkney and Klerksdorp, uranium from Klerksdorp, platinum from Rustenburg and Brits, and diamonds from Lichtenburg, Christiana, and Bloemhof. The province accounts for 20% of South Africa’s mining industry, and over 90% of the country’s platinum comes from its mines. The province is also renowned for cattle farming, particularly in areas around Stella (North West Province 2015).

### Study design

The study utilised the Within-Stage Mixed Model (Johnson & Onwuegbuzie 2004) for both quantitative (QUANT) and qualitative (qual) data. Data collection, analysis, and interpretation were triangulated, and closed-ended and open-ended questions were strategically combined. Quantitative data were analysed statistically, while qualitative data were analysed thematically. Themes from qualitative data supplemented or elaborated on responses to closed-ended questions.

### Study population and sampling procedure

The study targeted veterinarians and animal health technicians (AHTs) employed by the South African Government in the Sub-Directorate of the Animal Health, Directorate of Veterinary Services, North West Provincial Department of Agriculture and Rural Development (DARD). The target population was 141 individuals at the time of inquiry, and 53 participants were sampled using a non-probability sampling technique (convenience sampling) (Etikan et al. 2016) because of its low cost and availability of the target population in certain governmental events. Data were collected at various locations across the North West province including the South African Association for Animal Health Technicians (SAAAHT) North West Provincial Congress 2016, three State Veterinary offices, and the Provincial Rabies Day campaign.

### Data-collection tools and techniques

A self-administered questionnaire was designed with closed-ended and open-ended questions adapted from Adjei (2013) and Sabarinathan Nair, Rao and Ramkumar (1997). Validity was pre-tested. Closed-ended questions had multiple-choice questions and a 5-point Likert scale. Open-ended questions were for participants to clarify or complement responses provided to close-ended questions.

### Data analysis

IBM-Statistical Package for Social Science version 24.0 (International Business Machines Corporation (IBM), Armonk, New York State, United States [US]) was used to analyse quantitative data. Descriptive statistics were generated, including frequencies, percentages, mean, and standard deviations of variables. The Chi-square test determined associations between demographic characteristics and two variables that determined awareness and perceptions. However, the inferential statistics performed on the data are only applicable to the sample in this study as non-probability sampling was used in the study. A thematic analysis was conducted on the qualitative data to generate themes following the framework proposed by Braun and Clarke (2006), and the themes were then reported in narratives.

### Validity

The tool’s validity was tested by piloting it at North-West University’s Mafikeng campus. The pilot study included eight veterinarians, three AHT participants from the Centre of Animal Health Studies, and two staff members from the Department of Agricultural Extension and Economics who had expertise in questionnaire design. Feedback from the participants was used to determine the content validity of the questionnaire. After scrutinising and responding to the questionnaire, participants had one-on-one sessions to provide feedback on the tool. The instrument was revised based on their recommendations.

### Ethical considerations

The Institutional Research Ethics Regulatory Committee of the North-West University approved the research (Ethics number: NWU-00554-16-A9). The research proposal was submitted to the Chief Director of Veterinary Services of the North West Provincial Department of Agriculture and Rural Development (DARD) for permission to collect data among state-employed animal healthcare practitioners (AHCPs). An agreement was reached with the Chief Director of Veterinary Services that data can be collected after informed consent was obtained from AHCPs who were willing to participate. The AHCPs received an introductory letter attached to the self-administered questionnaire, explaining the details and purpose of the study. Participants were informed that participation was voluntary and completing and submitting the questionnaire indicated informed consent to participate. The data collection process respected the participants’ rights.

## Results

### Demographic characteristics of participants

A total of 53 participants took part in the study. Males comprised the majority at 56.6% (Table 1). The most common age range was 30–40 years at 32.1%. A total of 79.2% of the participants were AHTs, with 92.5% of the sample being black people, and 37.7% employed in the Ngaka Modiri Molema District Municipality. All four district municipalities in the North West province were represented in the sample.

**TABLE 1:** Demographic characteristics of participants.

Demographic characteristics	Descriptions	Frequency	%
Gender	Male	30	56.6
	Female	23	43.4
Age (years)	< 30	9	17.0
	30–40	17	32.1
	41–50	13	24.5
	51–60	14	26.4
Profession	Veterinarians	11	20.8
	Animal Health Technicians	42	79.2
Race	Black people	49	92.5
	White people	2	3.8
	Mixed race people	1	1.9
	Asian people	1	1.9
Professional experience in years	Less than 5	12	22.6
	Less than 10	13	24.5
	Less than 15	10	18.9
	Less than 20	8	15.1
	Less than 30	6	11.3
	Above 30	4	7.5
District municipality of employment	Ngaka Modiri Molema	20	37.7
	Dr Kenneth Kaunda	6	11.3
	Bojanala	11	20.8
	Dr Ruth Segomotsi Mompati	16	30.2

### Awareness and perceptions of animal healthcare practitioners on the use of ethnoveterinary medicine by farmers

**FIGURE 2:**
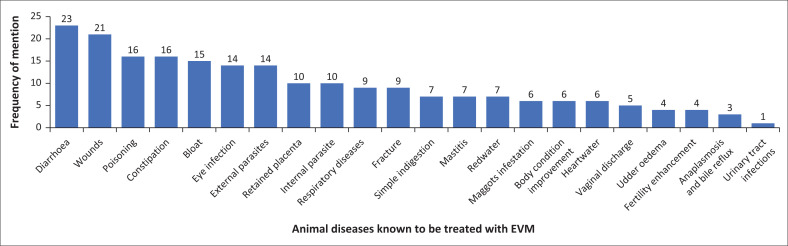
Frequency distribution chart of animal diseases known to be treated with ethnoveterinary medicine in farming communities (*n* = 41).

A majority at 77.4% (*n* = 41/53) of AHCPs in the study were aware of ethnoveterinary medicinal practices. There was no significant difference between awareness among veterinarians (72.7%) and AHTs (78.6%). Participants were aware of several diseases treated using EVM including, diarrhoea (*n* = 23), wounds (*n* = 21), poisoning (*n* = 16), and constipation (*n* = 16) as shown in Figure 2. The use of EVM by farmers was attributed to its local availability and affordability (56.10%), as well as the lack of access to Western veterinary services (41.46%) and the ease of application (36.59%) as shown in Table 2. However, most practitioners did not believe that farmers use EVM because of its effectiveness as only 12.2% indicated that farmers use EVM because of its effectiveness.

**TABLE 2:** The perceived reasons for use of ethnoveterinary medicine by farmers and the sources of information on ethnoveterinary medicine for Animal health care practitioners.

Variable	Reasons for use of EVM	Cases	Proportional percentage of responses (%)†
Reasons for use of EVM to farmers as perceived by Animal Healthcare Practitioners (*n* = 41)	Cheap and economical	23	56.1
	Locally and easily available	23	56.1
	Where Western veterinary assistance is not available	17	41.5
	Easy application	15	36.6
	Satisfies the owner	14	34.2
	Technical person not required	10	24.4
	Treatment at farmers’ residence	9	21.9
	Less toxic	5	12.2
	Effective	5	12.2
	Helps when animal is immobile	5	12.2
	Hastens the natural process of healing	4	9.8
	No side effects	3	7.3
	Less risk	1	2.4
	None of the above	1	2.4
Percentage distribution (%) of sources of EVM information for Animal Healthcare Practitioners (*n* = 41)	From community members (including farmers)	29	70.7
	From family members	16	39.0
	Never heard of it before now	12	29.3
	At conferences	8	19.5
	From colleagues	6	14.6
	On publications	5	12.2
	From traditional healers	5	12.2

EVM, ethnoveterinary medicine.

† , The percentage is calculated from cases that were more than the sample size as participants provided multiple responses.

### Sources of information on ethnoveterinary medicine for animal healthcare practitioners

The sources of information on EVM for AHCPs were identified in the study. The findings showed that indigenous communication networks, such as farmers, community members, and family members, were the primary source of awareness about EVM among AHCPs, as indicated in Table 2. Among these networks, community members (including farmers) were the most significant source of information (70.7%), followed by family members (39.0%).

### Approval of the use of ethnoveterinary medicine by farmers

The study aimed to gauge approval of EVM use by farmers using a 5-point Likert scale. The majority approved the use of EVM by farmers with a mean of 3.57 (standard deviation [s.d.] = 1.016) on the agreement scale which ranged from 1 (not in agreement) to 5 (in agreement). The highest percentage of participants (57.4%) approved the use of EVMs by farmers, while 29.8% were not sure if they support the use of EVM by farmers and 12.8% totally opposed the use of EVM by farmers.

### Themes emerging for reasons of approval or lack thereof for the use of ethnoveterinary medicine by farmers

Thematic analysis of the narrative by AHCPs to explain the reasons they approved or opposed the use of EVM by farmers identified four main themes, two against (themes 1 and 2) and two in favour (themes 3 and 4). Themes identified are as follows: (1) Ethnoveterinary medicine is unscientific; (2) Use of EVM interferes with Western veterinary services; (3) Ethnoveterinary medicine is an alternative or complementary to Western veterinary medicine; and (4) Ethnoveterinary medicine is effective. Theme 1 and 2 will be presented first.

#### Theme 1: Ethnoveterinary medicine is unscientific

The results revealed that participants who do not agree with the use of EVM believe that EVM has no scientific bases compared to Western veterinary medicine which is based on science. Under this main theme, two sub-themes emerged that exposed why participants consider EVM as unscientific.

Sub-theme 1: No scientific testing to determine the pharmacological properties and effects of EVM.

Participants believe that as EVM is indigenous knowledge, it lacks scientific testing and, therefore, lacks dosages, information on side effects, and withdrawal periods. The following narrative can best represent the views of participants: ‘Pharmacodynamics and pharmacokinetics of such remedies need to be scientific and published’ (Participant 2, male, Veterinarian). Therefore, participants would not recommend the use of EVM and this sentiment is best represented by the following excerpt: ‘I can only recommend medicines that fall in my jurisdiction or profession. That is, declared usable by pharmaceutical companies’ (Participant 21, male, Animal Health Technician).

Sub-theme 2: EVM is dangerous and toxic.

The sub-theme can best be summarised by the following statements: ‘Lots of the items used are toxic substances with more potential to harm than do good’ (Participant 15, female, Animal Health Technician) and also ‘Most medicines used have no known active ingredient[*s*], may have severe side effects because of other ingredients since the medicine is not purified’ (Participant 4, male, Veterinarian).

#### Theme 2: Use of ethnoveterinary medicine interferes with Western veterinary services

Participants believe using EVM delays consultation with AHCPs, leading to spread of zoonotic diseases and other serious diseases. This view is best represented by the following comment:

‘But I feel modern medicine is constantly improving and is more effective and a vet can examine an animal, give the correct diagnosis and ensure that the correct medication is provided to prevent the spread of diseases.’ (Participant 50, male, Veterinarian)

Participants believed that if farmers implement EVM, they will reject Western veterinary services. This view is summarised by the following excerpt, ‘Farmers resist the use of correct medicine’ (Participant 3, male, Animal Health Technician).

#### Theme 3: Ethnoveterinary medicine is an alternative or complementary to Western veterinary medicine

Participants suggest that EVM serves as an alternative or complementary to Western medicine, especially when veterinary services are unavailable or costly. The following excerpts best represent this perspective, ‘It could be that farmers are far from co-ops [*co-operatives selling veterinary drugs and remedies*] or town to buy vet medicine or maybe they are struggling with finances’ (Participant 11, male, Animal Health Technician) and also ‘In remote areas, Animal Health Technicians are scarce’ (Participant 6, male, Animal Health Technician).

#### Theme 4: Ethnoveterinary medicine is effective

Participants believe that EVM is effective for treating ailments in animals, as it has survived the test of time and has been testified by farmers. This belief is best expressed in the following excerpts, ‘Farmers have been using EVM for generations and were never disappointed; otherwise, they could have quit using the medicines’ (Participant 49, male, Animal Health Technician), ‘In the past decades, medicines were not there but our grandparents and great-grandparents managed to cure animals with herbs, it is beneficial’ (Participant 18, male, Animal Health Technician) and ‘Farmers will always tell me stories about their successes with the use of ethnoveterinary medicine’ (Participant 16, female, Animal Health Technician).

### Perceptions on effectiveness and limitations of ethnoveterinary medicine

**FIGURE 3:**
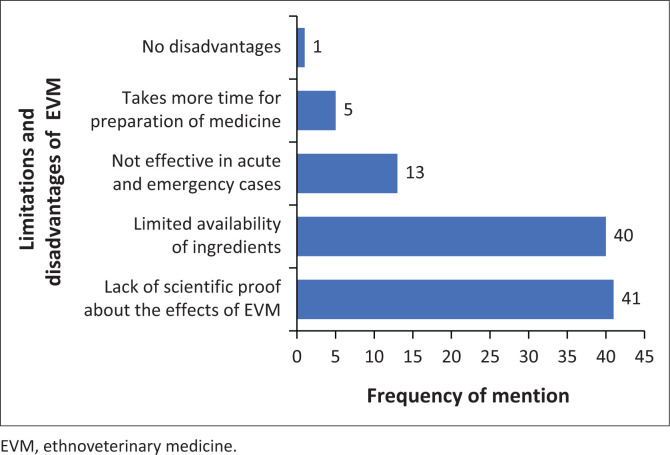
Frequency distribution chart of the perceived limitations and disadvantages of ethnoveterinary medicine (*n* = 41).

The study found that most participants (65.8%) were uncertain about the effectiveness of EVM. However, a smaller percentage (22.0%) believed that EVM was effective, while only 12.2% perceived it as ineffective. The study also revealed that the lack of scientific proof (*n* = 41/41) and limited availability of ingredients (*n* = 40/41) were the greatest limitations of EVM as an alternative or complementary to Western veterinary medicine as indicated in Figure 3.

### Use of ethnoveterinary medicine among animal healthcare practitioners in the North West province

According to the study, 20.8% (*n* = 11/53) of AHCPs use EVM to treat a total of 15 diseases. The diseases were, retained placenta, internal parasites, gall sickness (bile reflux), eye infection, metritis, fracture, poisoning, external parasites, constipation, bloat, wounds, diarrhoea, heartwater, redwater, and lumpy skin disease. Participants used EVM either because they trusted the remedies (54.5%) or were experimenting (36.4%) or when modern medicine failed (18.2%) or took too long to heal (27.3%), or when farmers requested it (27.3%).

### Recommendations for the improvement of ethnoveterinary medicine by Western-trained animal health practitioners

**FIGURE 4:**
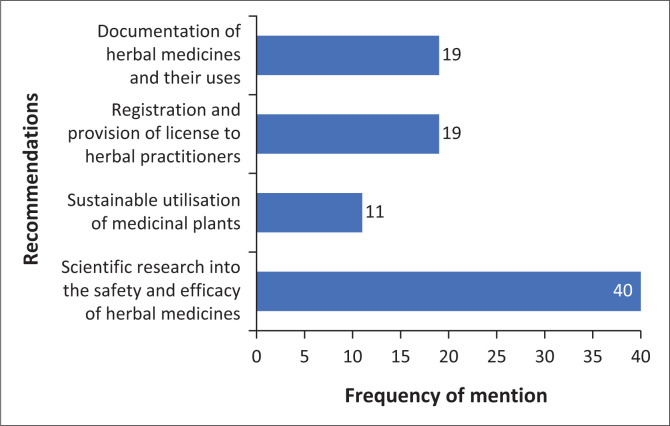
Frequency distribution chart of recommendations by participants for the improvement of ethnoveterinary medicine (*n* = 41).

As shown in Figure 4, participants recommend scientific testing of EVM for safety and efficacy (*n* = 40/41). They also suggest documenting EVM plants and their uses, (*n* = 19/41) as well as providing licenses for traditional medicine practitioners (*n* = 19/41).

### Inferential statistics

The Chi-square test revealed an association between the district of employment and awareness of EVM (H_a_5). The null hypothesis was rejected at χ^2^ = 17.490, *df* = 3, and *p* = 0.001. Table 3 displays that AHCPs at Dr Ruth Segomotsi Mompati had the highest awareness of EVM (100%), while those at Dr Kenneth Kaunda had the lowest (16.7%). The Chi-square test revealed no associations between AHCPs’ agreement with the use of EVM and their gender, age, profession, or professional experience.

**TABLE 3:** Cross-tabulation of district municipality of employment and awareness of ethnoveterinary medicine among participants to determine association between the two variables.

Variable	Responses	District municipality				Total
		Ngaka Modiri Molema	Dr Kenneth Kaunda	Bojanala	Dr Ruth Segomotsi Mompati	
Have you heard or experienced EVM?	No (%)	25.0	83.3	18.2	0	22.6
	Yes (%)	75.0	16.7	81.8	100.0	77.4

**Total (%)**	-	-	-	-	-	**100.0**

EVM, ethnoveterinary medicine.

## Discussion

### Awareness of ethnoveterinary medicine among Western-trained animal health practitioners

The majority of AHCPs in this study are aware of ethnoveterinary medicinal practices used by farmers. The study dispels the notion suggested by Toyang et al. (1995) that EVM is practised in secret. The high level of awareness among AHCPs in the North West province indicates the presence of a rich amount of ethnoveterinary knowledge among farmers and communities, as well as a willingness to share this knowledge. However, the smaller percentage of AHCPs who are unaware of EVM could be linked to their location of employment (rural or urban), and type of farming (commercial or communal). Ethnoveterinary medicine is mostly practised among rural and/or poor farmers (Luseba & Van der Merwe 2006; Masika, Van Averbeke & Sonandi 2000; McGaw & Eloff 2008), indicating that AHCPs stationed in commercial farming areas or urban areas are likely to be uninformed about EVM.

The study found that participants employed in Ngaka Modiri Molema, Bojanala, and Dr. Ruth Segomotsi Mompati District Municipalities had higher awareness of EVM practices compared to those in Dr Kenneth Kaunda. The location and economic profiles of the districts influenced the study’s findings. Most AHCPs in Dr Kenneth Kaunda are based in urban state veterinary offices in economic hubs like Potchefstroom and Klerksdorp. This suggests that they provide services to urban, peri-urban, and commercial farmers who do not use EVM. On the other hand, Dr Ruth Mompati, the most rural and poor district in the province, had all AHCPs aware of EVM. The socio-economic characteristics of the district have resulted in the communities’ reliance on EVM instead of expensive Western medicine, which explains the level of awareness of EVM practices among Western-trained animal health practitioners.

The results of this study when compared with Indian studies conducted by Jirli, Jha and Chugh (1997) and Sabarinathan Nair et al. (1997) show that AHCPs in India are more proficient in EVM issues than the participants in this study. In a study conducted by Jirli et al. (1997), 15 AHCPs were evaluated for their knowledge and acceptance of EVM at the National Dairy Research Institute in Karnal, Haryana. The participants were veterinarians and almost all were aware and over half of them demonstrated practical knowledge of EVM for specific animal health treatments. Sabarinathan Nair et al. (1997) conducted a survey on ethnoveterinary medicinal practices in Pondicherry, Tamil Nadu, and Kerala states. The findings showed that over three-fifths of AHCPs used EVM to treat various ailments. The study by Jirli et al. (1997) and Sabarinathan Nair et al. (1997) found higher awareness and knowledge in EVM in India compared to the South African counterparts in this current study, likely because of India’s more established EVM system and research (Hoareau & DaSilva 1999). In addition, in India it is common practice for AHCPs to integrate EVM with Western medicine (Sabarinathan Nair et al. 1997).

The study found that indigenous communication networks are the main source of knowledge for AHCPs regarding EVM, while Western communication networks like conferences play a minor role. This finding is similar to that of Jirli et al. (1997), who discovered that indigenous communication networks were the primary EVM knowledge source for participants. Thus, the current study revealed a gap in ethnoveterinary research and development in South Africa, specifically in sharing information at conferences attended by para-veterinarians and veterinarians. The researcher suggests promoting the new field of EVM extensively, making use of all available opportunities, including conferences, and targeting mainly AHCPs.

### Ethnoveterinary medicine is tolerated but its value is not appreciated

Individual perception determines acceptance of any phenomenon (Ogunniyi 2007). In this study, a moderate majority of AHCPs agreed with using EVM in farming. Similar findings among Western-trained healthcare practitioners (WTHCPs) in Limpopo and Gauteng provinces in South Africa were reported by Mokgobi (2012). The majority of WTHCPs accept traditional medicine as a primary healthcare system. Similarly, in the current study, some participants believe EVM is a good primary animal health system, especially for poor-resourced farmers and those in remote areas. Moderate agreement with traditional medicine use, including EVM, found in this and other studies including Mokgobi (2012) are positive predictors for government-suggested integration of Western and traditional medicine.

An interesting finding of this study is that although most participants agreed with the use of EVM by farmers, a higher number were uncertain about its effectiveness. This suggests that AHCPs tolerate EVM despite not seeing its value. The reason for this could be that most participants perceive EVM as lacking a scientific basis and, therefore, do not trust in its effectiveness. These concerns coincide with those of similar studies in either ethnomedicine or the field of ethnoveterinary by Sabarinathan Nair et al. (1997), Fielding (1997), Jirli et al. (1997), Hopa et al. (1998), Adjei (2013), Peltzer, Mngqundaniso and Petros (2006), Van Rooyen et al. (2015), Peu (2000), and Mokgobi (2012).

It is understandable why AHCPs would be worried about the lack of scientific testing, as this study points out. Their concerns are not without merit. A review of EVM in South Africa (McGaw & Eloff 2008) reported that majority of documented medicinal plants were not tested at the time and recommended further validation testing for the majority of the plant species. However, the lack of scientific validation for EVM is not limited to South Africa; Iqbal and Jabbar (2010) found the same trend in their global review of EVM research. Ethnoveterinary research and development is a new field in South Africa and the progress or lack thereof can be attributed to its early stages of development. However, significant research has been conducted on medicinal plants and their scientific validation as evident in South African reviews by McGaw and Eloff (2008), Chakale, Mwanza and Aremu (2021); Selogatwe et al. (2021). The uncertainty among AHCPs regarding the effectiveness of EVM may be because of a gap in the dissemination of information on ethnoveterinary research and development.

Another interesting finding was that some AHCPs also practice EVM, often because of trust in the practice. How and when did they learn to appreciate EVM? Their appreciation for EVM may have developed in childhood or later in life, influenced by sources such as parents or cultural upbringing, and shaped by past experiences or socio-cultural settings (Ellen, Lycett & Johns 2013; Kashyap 2016). These factors can contribute to the formation of positive perceptions of EVM, leading to its practice.

## Conclusion

The study examined the awareness and perceptions of EVM among AHCPs. Results showed high awareness and moderate tolerance, but most participants doubted its effectiveness and perceived it as unscientific. The main reason for the lack of trust in medicinal plants is the perception that they have not been thoroughly tested to establish their safety and effectiveness. However, many medicinal plants in South Africa have been scientifically validated. The issues of lack of standardisation and regulation may also contribute to this lack of trust. The undervaluing of the benefits of EVM as a sustainable and complementary alternative to Western medicine could hinder the integration of traditional and Western medicine practices in South Africa. The study recommends promoting EVM among stakeholders through conferences and workshops to educate AHCPs on the latest scientific research and developments in the field of EVM.
